# Emerging New Approaches in Desensitization: Targeted Therapies for HLA Sensitization

**DOI:** 10.3389/fimmu.2021.694763

**Published:** 2021-06-11

**Authors:** Ashley Y. Choi, Miriam Manook, Danae Olaso, Brian Ezekian, Jaeberm Park, Kyle Freischlag, Annette Jackson, Stuart Knechtle, Jean Kwun

**Affiliations:** Duke Transplant Center, Department of Surgery, Duke University Medical Center, Durham, NC, United States

**Keywords:** sensitization, desensitization, alloantibody, plasma cells, germinal center

## Abstract

There is an urgent need for therapeutic interventions for desensitization and antibody-mediated rejection (AMR) in sensitized patients with preformed or *de novo* donor-specific HLA antibodies (DSA). The risk of AMR and allograft loss in sensitized patients is increased due to preformed DSA detected at time of transplant or the reactivation of HLA memory after transplantation, causing acute and chronic AMR. Alternatively, *de novo* DSA that develops post-transplant due to inadequate immunosuppression and again may lead to acute and chronic AMR or even allograft loss. Circulating antibody, the final product of the humoral immune response, has been the primary target of desensitization and AMR treatment. However, in many cases these protocols fail to achieve efficient removal of all DSA and long-term outcomes of patients with persistent DSA are far worse when compared to non-sensitized patients. We believe that targeting multiple components of humoral immunity will lead to improved outcomes for such patients. In this review, we will briefly discuss conventional desensitization methods targeting antibody or B cell removal and then present a mechanistically designed desensitization regimen targeting plasma cells and the humoral response.

## Introduction

Desensitization treatment within the field of transplantation refers to the process of antibody removal (1), specifically preformed donor-specific HLA antibody (DSA). DSA as a barrier to successful transplantation was first described in early reports of kidney hyperacute rejection in the context of positive complement dependent cytotoxicity (CDC) by Ramon & Terasaki et al. (1), Improvements in histocompatibility testing have removed the risk of hyperacute rejection across all organs, yet acute and chronic AMR remain a major contributor to poor transplant outcomes (2, 3).

### The Current Status of HLA Sensitization

For many patients awaiting transplantation, blood transfusion, prior transplantation, and pregnancy are the sources of sensitization (4, 5). There is evidence that the primary source of HLA sensitization is important. Transplantation appears consistently as the strongest sensitizing event inducing both class I and II HLA antibody (6, 7); however, there is some evidence that post-transplant, those with pregnancy induced HLA-antibody respond more rapidly (5). Waiting times for highly sensitized patients, calculated Panel Reactive Antibody (cPRA)> 80% (8) are longer, leading to increased morbidity and mortality. Compared to patients with absent or low cPRA, the highly sensitized candidates could expect to wait twice as long for a compatible transplant in both the USA (9) & UK (10). In Europe, the acceptable mismatch program has long been advocated to reduce waiting times for highly sensitized patients (11). Recently, in the USA the new kidney allocation scheme (KAS) (12), was specifically designed to improve the transplantation rates for sensitized patients by providing more allocation points and mandating regional and national sharing for those with the highest CPRA. Early KAS reports suggest that this has largely been successful (13, 14).

For sensitized patients with an incompatible living kidney donor, to whom they have DSA, the decision is whether to use a kidney paired donation (KPD) program to obtain a compatible match, await a compatible deceased donor offer, or proceed with a HLA-incompatible, positive cross match transplant (HLA-i) which requires desensitization prior to transplantation. Simulation of how KPD programs might run were initially optimistic (15), but over time many struggle with a pool enriched with highly sensitized patients unless specific matching interventions are made (16), or a combination of both desensitization and KPD is utilised (17).

### The Current Standing of Desensitization

In the USA, multicenter data demonstrates a clear survival benefit (18) in proceeding with an HLAi; however, in the UK the picture is more nuanced with no survival benefit demonstrated, although for the patients awaiting a compatible transplant, around 40% remain untransplanted at 5 years post-listing (19). Desensitization then remains a guaranteed route for a highly sensitized recipient to obtain transplant, albeit with greater immunological risk. To date, desensitization therapies have largely relied upon physical methods of antibody removal in the form of repeated plasmapheresis, in conjunction with additional agents. In this review, we will outline the currently used regimen for desensitization, as well as describing new potential approaches.

## The Current Desensitization Treatment Strategy

In highly sensitized patients removal of circulating anti-HLA antibody or lowering cPRA is an important and fundamental strategy for expanding donor options and successfully transplanting across DSA barriers. However, durable inhibition of HLA antibody production is the “holy grail” for successful kidney transplantation (KT) in sensitized patients.

### Plasmapheresis or Immunoadsorption

Plasmapheresis (PP) has been used for several decades as a method for lowering circulating antibody in various immune diseases (20, 21). Plasmapheresis physically removes large molecular weight substances from the plasma, including antibodies, complement components immune complexes and coagulation factors (22). Using the double filtration plasmapheresis (DFPP) system, a cascade of filtration traps larger molecules, and thus allow lower molecular weight components to pass back to the patient (23).Together with IVIg, it has been used to effect successful transplantation for positive crossmatch patients, and for many units, is the mainstay of desensitization prior to transplantation (24–26). In Europe and Australia immunoadsorption (IA) using staphylococcal protein A column has been applied in eliminating antibodies (27–29). The kinetics of antibody removal by PP are predictable within limited periods compared with other treatment modalities since plasma proteins are reliably removed (30). Therefore, PP or IA can be used as an effective treatment modality in the setting of planned transplantation across a positive HLA cross-match in living donor KT. PP or IA has a limitation of antibody rebound after the completion of treatment sessions.

### Intravenous Immunoglobulin

IVIG has been widely used in inflammatory and autoimmune conditions (31). IVIG also has a role in AMR treatment in kidney transplantation (32). Although widely used as part of desensitization regimens for many decades, the precise mechanism of action is unknown as a result of its broad spectrum of effects. Many potential mechanisms of action of IVIG in transplantation have been proposed. The main mechanisms are considered to be neutralization of circulating anti-HLA antibodies with anti-idiotypic antibodies (31), the inhibition of complement activation (33, 34), and binding to Fc receptors on immune cells (35, 36). It is also postulated that IvIg following plasmapheresis prevents rebound of DSA, by providing an abundant quantity of circulating IgG (37, 38). IVIG has been used in various doses according to protocol from 100mg/kg to 2.0g/kg in desensitization prior to living donor KT or for deceased donor KT of patients with high PRA.

Although various combinations of IVIG or PP with rituximab have been proposed, two protocols have been widely accepted and used (39).

### PP With Low-Dose IVIG vs. High-Dose IVIG Alone

Using PP with low-dose IVIG, many centers report transplant outcomes with acute AMR rates of 12-43% when used in combination with various induction agents, anti-thymocyte globulin, anti-IL-2Rc antibody or OTK3 (40–43). The NIH IGO2 study, a controlled clinical, multi-center, double blinded trial of IVIG (2g/kg, monthly 4 times) versus placebo in sensitized patients, HLA antibody levels were reduced further, and the transplantation rate was higher in the IVIG group than in the placebo group (44). Glotz et al. reported results of high-dose IVIG desensitization with anti-thymocyte globulin induction in cross-match positive patients (45). Jordan et al. reported successful transplantation outcomes with two doses of 2mg/kg IVIG on day 0 and day 30 with rituximab in 20 patients (46). In this study, 16 patients among 20 could receive KT within 6 months. In their subsequent series, they used high-dose IVIG 2g/kg, 3 times on day 1, day 30 and at the time of transplantation with rituximab (47). Among 76 patients with PRA ≥30%, 31 patients received living donor KT, and 45 patients received deceased donor KT with reduced waiting time of 4.2 ± 4.5 months.

### Anti-CD20 Antibody (Rituximab)

Rituximab is an anti-CD20 monoclonal antibody that binds to CD20 expressed on immature and mature B-lymphocytes, inducing apoptosis *via* antibody-dependent cytotoxicity, complement-dependent cytotoxicity or direct apoptosis. Originally, anti-CD 20 antibody was used to treat B-cell lymphoma. In transplantation, rituximab was introduced to deplete B cells with the goal of reducing donor-specific antibody (DSA) production (48). Rituximab has been used as an additional therapy as part of desensitization treatments, in conjunction with plasmapheresis & IvIg (46, 49). The half-life of rituximab in patients with end-stage renal disease is known to be 9-14 days (50). Rituximab administration can maintain durable B-cell depletion for at least six months, but rituximab does not bind to plasma cells as they do not express CD20 (51).

### Unmet Need For Sensitized Patients

Because no randomized controlled clinical trial has compared the two main protocols described above, and the study populations and the criteria for transplantation vary, it is difficult to evaluate which protocol is best. Desensitization protocols using high-dose IVIG or low-dose IVIG + PP with rituximab have relative advantages and disadvantages. A PP-based protocol with low-dose IVIG, within limited periods, is more effective and predictable for lowering antibody levels. On the other hand, in spite of possible non-response, high-dose IVIG has the advantage in patients with high PRA on the waiting list of being less invasive given the unpredictable time to transplantation. However, both current desensitization protocols have limitations. Regardless of whether high-dose IVIG or low-dose IVIG with PP were used, acute AMR rate as well as acute cellular rejection rates were higher in desensitized patients than in non-sensitized patients (52). In a study that included surveillance biopsy of desensitized KT recipients, the subclinical AMR rate was 31% at 3 months post-transplantation, and patients with subclinical AMR at 3 months post-transplantation had higher C4d, ptc and arteriosclerosis scores post-transplantation at 1 year than the patients without subclinical AMR at 3 months post-transplantation (53). Transplant glomerulopathy was reported at a rate of 44% at a mean of 18 months post-transplantation (54). After desensitization, long-term outcomes of KT seems to be worse than for unsensitized patients (42).

## Sensitization in Thoracic Organ Transplant Recipients: Similarities and Differences

Thoracic transplantation shares similar immunologic challenges as HLA sensitization in kidney recipients. However, no alternative organ replacement modalities support life in end-stage lung disease as dialysis in end-stage renal disease. While left ventricular assist devices (LVAD) have emerged as a viable alternative to heart transplant, it is not without significant risks and complications that limit access to therapy. As such, thoracic transplantation faces a greater urgency and waitlist mortality, and desensitization regimens must take into account these temporal challenges of sensitized patients. 1 in 7 adult heart transplant candidates are sensitized, a number that has doubled in the past two decades (55). A rising incidence is anticipated due to the expanding use of LVADs as a bridge to transplantation, advanced congenital heart disease surgery leading to more patients surviving to require transplant, and, to a smaller extent, an increase in re-transplantation (55). On the contrary, the true burden of sensitization in lung transplantation is unknown. National and international registries lack robust DSA or PRA/cPRA data for lung transplants. In the ISHLT registry, women, who are known to have greater sensitization secondary to pregnancy, comprise 60% of the waitlist but receive only 43% of the transplants, with a median time to transplantation of 233 days compared to 86 for men (56). To better understand and quantify this issue, comprehensive cPRA reporting in lung transplantation registries is required. Many lung transplant programs currently practice avoidance of DSA at the time of organ allocation, significantly limiting sensitized candidates’ access to transplant (57).

A common sequela of AMR in heart transplantation is cardiac allograft vasculopathy (CAV), and in lungs, chronic lung allograft dysfunction (CLAD), both of which result in significant mortality and morbidity within 5 years of transplantation (57–63). The primary goals of desensitization in thoracic transplantation are to increase access to transplantation through expansion of the donor organ pool and to prevent AMR and its subsequent morbidity and mortality. No approach has demonstrated significant and sustainable reductions in HLA antibody prior to transplant, and patients with elevated PRA continue to be at higher risk for rejection and reduced survival (64).

### Shifting Toward Sensitization

As mentioned, use of mechanical support as bridge to transplantation has been steadily increasing, reaching 50% of patients on the waiting list for heart transplant in 2013. Particular attention to the immunologic challenges associated with LVADs to target interventions is necessary. While many studies suggest that LVAD-associated allosensitization limits sensitized candidates’ access to transplant, they fail to show that it leads to rejection or increased mortality after receiving a transplant (65). Notably, most of the evidence implicating such findings has been gathered from studies that examined pulsatile-flow LVADs and pre-dates the use of current generation continuous-flow LVAD (65–67). In a more recent study by Ko et al, 23% of patients became newly sensitized after continuous-flow LVAD implant (68). Compared with patients without new sensitization or those already sensitized at baseline, these patients had an increased risk of ACR and AMR, but comparable survival 5 years post-transplant, consistent with an earlier study (69). This suggests that even if the alloantibody levels were decreased before transplant by conventional methods, such as IVIg and plasmapheresis, maintenance immunosuppression targeting memory B cells and plasma cells is critical to prevent rebound DSA. In addition, the patients who were newly sensitized after LVAD implant and did not reach transplantation had a higher level of allosensitization (27.9% vs. 10.2%) and a high mortality of 39.5% during follow-up. This is consistent with a study by Alba et al. that also found an association between high PRA and lower transplant probability that likely drives the high mortality observed (70). A key concern with LVAD is the requirement of blood transfusions that result in the generation of new anti-HLA antibodies; however, our understanding of the mechanism by which patients on LVAD support develop allosensitization is largely unchanged since 1999 (71). It is also known that platelets and fibrinogen can adhere to the surface of LVAD coated with polyurethane membrane and form a fibrin matrix which traps other cells (72). The trapped cells could provide subsequent excessive activation signals *via* cytokines and costimulation to T cells. During this aberrant state of T cell activation, LVAD patients are believed to develop B cell hyper-reactivity with subsequent allosensitization. By way of a CD95-dependent pathway, these activated T cells then undergo apoptosis (73).

### Current Desensitization Strategy in Thoracic Organ Transplantation

Despite these challenges, efforts to desensitize patients on the waitlist have generated limited success. The current research is centered around renal transplant experience with application in thoracic transplantation limited by several factors. In both heart and lung transplantation, there are requirements for donor-recipient size matching and transplant urgency is comparatively greater. Consequently, patients do not survive to begin clinical trials and the unpredictable nature of donor availability significantly limits the use of desensitization treatments prior to transplantation as prolonged period of treatment may confer more risks than benefits. Progress has been made from using IVIg and PP alone to using a variety of targeted therapies, although evidence in thoracic transplantation remains scarce. No large cohort desensitization strategy has been described in thoracic transplantation.

## New Pharmacologic Strategies for Desensitization

### Targeting Antibodies

#### IgG Endopeptidases

More recently, attempts have been made to fundamentally alter the structure of preformed antibody, using IgG endopeptidase (IdeS) which is a bacterial enzyme produced by *S. pyogenes* that cleaves all four human IgG subclasses into F(ab) & F (c) fragments, thus inhibiting both complement-dependent cytotoxicity and antibody-dependent cytotoxicity (74). IdeS has additional effects by cleaving the IgG present in the B-cell receptor complex (BCR), thus switching off B-cell memory as a downstream effect (75). Jordan et al. recently completed a trial of IdeS in 25 highly sensitized patients prior to HLA-incompatible kidney transplantation (76). All patients had near-complete or complete reductions of anti-HLA antibodies and donor-specific antibodies at 24 hours post-transplant, which allowed successful transplantation in 24/25 (96%). However, in 1-2 weeks the levels of these antibodies rebounded. Ultimately, one patient had graft loss from hyperacute rejection, while 10/25 (40%) had evidence of antibody-mediated rejection in the early post-transplant period. These findings suggest that IdeS has strong, albeit transient, ability to reduce DSA that may make this therapy useful in combination with strategies that allow for longer-term control of DSA rebound.

#### Anti-FcRn Approach

Brambell et al. identified FcRn, a neonatal IgG receptor that is closely related to the MHC Class I receptor, which is involved in a variety of critical biological and immunological functions, most notably regulating serum IgG levels and the recycling and transcytosis process that results in an increased half-life of IgG and albumin in human serum (77–80). Strategies that block the IgG-FcRn interaction are hypothesized to promote IgG degradation and decrease pathogenic autoantibodies and alloantibodies (81, 82). IVIG was one of the first therapies to decrease anti-HLA antibodies and treat antibody-mediated autoimmune diseases through blocking the IgG-FcRn pathway, leading to saturation of FcRn receptors and degradation of IgG molecules (78, 83, 84). Since then, multiple therapies targeting FcRn or the IgG-FcRn interaction have been developed as treatment for autoimmune and infectious diseases, with promising benefits as therapeutic agents in reducing AMR in transplantation. Several monoclonal antibodies against FcRn such as M281, SYNT001, Rozanolixizumab, RVT-1401, and ABY-039 are in various clinical development stages. M281, a deglycosylated IgG anti-FcRn mAb, was well tolerated and achieved reduction of serum IgG levels of 80% from baseline in a phase I clinical trial (85). Rozanolixizumab (UCB7665) is a high affinity anti-human neonatal FcRn mAb that reduced plasma IgG concentrations in cynomologus monkeys by up to 85% (86). This led to a Phase I clinical trial of Rozanolixizumab in healthy human subjects that demonstrated therapeutic potential with sustained dose-dependent reductions in serum IgG concentrations when administered IV or SC (87). Phase II clinical trials of Rozanolixizumab were recently completed in patients with immune thrombocytopenia (NCT02718716) and myasthenia gravis (NCT03052751) (86). Seijsing et al. found that an engineered alternative scaffold protein [affibody molecule (Z_FcRn_)] effectively blocked the IgG-FcRn interaction when repeated injections of Z_FcRn_ and Z_FcRn_ fused to an albumin binding domain (ABD) in mouse models led to a 40% reduction of IgG in serum (88). ABY-039 is a molecule similar to Z_FcRn_ -ABD undergoing phase I trial (NCT03502954). Additional studies in animal models that inhibit IgG-FcRn binding include an anti-FcRn directed mAb, 1G3, that accelerated endogenous serum IgG clearance and reduced the severity of myasthenia gravis in rat models (89). Abdegs, an engineered antibody that inhibited FcRn recycling and enhanced IgG degradation, was efficacious in a murine model of arthritis (90). Synthetic FcRn-binding peptides (FcBP), small molecule FcRn antagonists, and other molecules that interact with the Fc binding site may also block IgG-FcRn interactions (78). Our group also tested anti-rhesus FcRn mAb in a skin-sensitized NHP model with kidney transplantation (*manuscript in submission*). Treatment with aFcRn prior to transplantation significantly reduced the levels of total and donor-specific alloantibody. However, in the context of renal transplantation, anti-FcRn treatment did not block the synthesis of DSA, such that transient reduction in DSA was followed by robust DSA increase and antibody-mediated rejection (*manuscript in submission*). The anti-FcRn approach demonstrated promising applications in lowering alloantibody levels in transplantation; however potential limitations and complexity of using the agent require further investigation in transplantation.

### Targeting Plasma Cells

Following the discovery that alloantibody secreting cells predominantly exist as long-lived plasma cells (LLPC) in the bone marrow compartment, along with the identification of these cells as being CD138^+^CD20 (91), bortezomib was used to lower alloantibody (92). Bortezomib, a proteasome inhibitor (PI) which depletes non-malignant plasma cells, was proposed to reduce anti-donor HLA antibody. While some groups have demonstrated efficacy of bortezemib to desensitize transplant recipients, the drug was used in combination with conventional therapies (93). Now several biologics targeting plasma cells are available and are being considered.

#### Targeting Plasma cells with Proteasome Inhibition

Bortezomib (Velcade^®^) is a 1^st^ generation, reversible inhibitor of the 26S proteasomal subunit. This drug is a potent inhibitor of plasma cells, which rely on rapid protein turnover to continually secrete antibodies, and succumb to oxidative stress and apoptosis when cellular recycling mechanisms are rendered nonfunctional. For this reason, bortezomib is approved for usage in multiple myeloma, a malignancy of plasma cells (94). Everly et al. first described its use as effective treatment of AMR and ACR as well as reduction in DSA in kidney transplant recipients (95), and Mulder et al. showed that proteosome inhibitors bortezomib, carfilzomib, oprozomib (ONX 0912), and immunoproteasome inhibitor ONX 0914 (previously PR-957) reduced B-cell proliferation, immunoglobulin production, and induced apoptosis of activated B-cells (96). Following some success for usage in refractory antibody-mediated rejection after kidney transplantation (97, 98), several groups have used bortezomib in the context of desensitization. Woodle et al. in the first trial with bortezomib variably combined with plasmapheresis and rituximab showed modest success with a reduction in the immunodominant DSA of 38/44 (86%) highly sensitized patients, successful transplantation of 19/44 (43.2%), and 17/19 (89.5%) of grafts functional at a median follow-up of 436 days (92). Jeong et al. used a combination of high dose IVIG, rituximab, and bortezomib and demonstrated a small reduction in the MFI value of class I PRA, and an increased rate of deceased donor kidney transplantation (8/19 or 42.1% of desensitized patients vs. 4/17 or 23.5% of controls, p = 0.004) with no graft loss in the desensitized group at a median follow-up of 23 months (93). The interpretation of these early favorable outcomes was limited by the small, non-randomized nature of the studies, and the confounding nature of its combination with conventional desensitization methods. Studies using bortezomib as monotherapy for desensitization have shown less promising results with poor reduction of anti-HLA antibodies and significant toxicity with longer courses of the drug (99, 100) that have caused enthusiasm for its use in new desensitization regimens to wane.

Carfilzomib (Kyprolis^®^) is a 2^nd^ generation, irreversible inhibitor of the 20S proteasomal subunit. Studies in patients with multiple myeloma suggest that this drug may be more efficacious and better tolerated than its predecessor bortezomib (101). A current clinical trial of carfilzomib for desensitization is underway (NCT02442648). Most recently, carfilzomib was studied as desensitization monotherapy yielding 72.8% median reduction in HLA antibodies and a 69.2% reduction in bone marrow plasma cells with acceptable drug safety and toxicity (102). Another second generation PI, ixazomib, warrants further testing. Ixazomib is an oral-form peptide boronic acid proteasome inhibitor distinct from bortezomib and recently had a successful phase III trial (TOURMALINE-MM1) in multiple myeloma (103–106). Other PI’s including marizomib, delanzomib, and oprozomib are being studied as anti-cancer and autoimmune therapies. PI’s have notably been studied most recently as desensitization therapy and additional studies in utilizing PI as maintenance immunosuppressive treatment are needed.

#### Immunoproteasome Inhibitors

Conventional PIs are broad spectrum PIs with various dose-dependent adverse effects. An attractive alternative would be to solely target the proteasome of immune cells. Hematopoietic origin cells display proteasomes with distinct catalytic subunits and the complex is referred to as the immunoproteasome (107). Interestingly, immunoproteasome is also expressed in nonhematopoietic cells exposed to pro-inflammatory mediators such as IFN-γ and TNF-α (108). Therefore, inhibition of the immunoproteasome allows for both the targeting of immune-specific cells but also cells actively involved in the inflammatory response. In kidney transplantation, it was found that patients with chronic AMR have up-regulated immunoproteasome activity (109). Newly developed immunoproteasome inhibitors (IPI) could selectively inhibit proteasomes of cells involved in graft rejection after transplantation, such as B and T lymphocytes and APC’s, and regulate pro-inflammatory cytokines and the differentiation of helper T cells (110, 111). But similar to conventional PIs, PC population would be more sensitive on IPIs. Current work in animal models has found that IPI is superior to PI in suppressing the cellular and humoral immune response, preventing chronic AMR, and prolonging survival (110–112). ONX-0914, formerly known as PR-957, is an LMP7-selective immunoproteasome inhibitor that is undergoing clinical studies in the treatment of autoimmune diseases and has potential applications in transplantation (110, 111). ONX-0194 and bortezomib combined suppressed DSA production, B cells and plasma cells after kidney transplant, inhibited IgG, complement, and proinflammatory cytokines IFN-y and IL-17, and reduced chronic allograft nephropathy. In mismatched mouse cardiac transplantation, IPI treatment with a noncovalent LMP-7 inhibitor, DPLG3, combined with CTLA4-Ig led to decreased effector T cells and T cell exhaustion (113). Other IPI’s such as Ipsi-001 and PR-924 are currently under investigation as potential anti-cancer agents. IPI is particularly attractive due to its specificity on immune cells which shows larger safety margin compared to conventional immunoproteasome inhibitors (114, 115). This may allow the continuous (or long-term) treatment of IPI after transplantation in sensitized recipients.

Outside of multiple myeloma therapies, there is still a range of opportunities to target alloantibody reduction. Building on the success of PIs, there has been focus on inhibiting protein degradation *via* inhibition of initial ubiquitin binding rather than the downstream proteasome complex (116). Another promising avenue is modulating the endoplasmic reticulum (ER). Inositol-requiring enzyme 1 (IRE1) inhibitors are currently under development and may be available in the near future (117, 118).

#### Monoclonal Antibodies for Targeting Plasma Cells

Inhibiting proteasome activity with PI should affect more than plasma cell population since all eukaryotic cells utilize proteasome to maintain their homeostasis. Even IPI should have broad impact on immune cells. Therefore, monoclonal antibody targeting of plasma cell population is very attractive.

CD38 is expressed at high levels by B lineage progenitors in bone marrow, B-lymphocytes in germinal centers, and terminally differentiated plasma cells (119, 120). Conversely, mature naive and memory B cells express low levels of the molecule (121, 122). Plasma cells (PC) actively producing allo-antibodies should express high levels of CD38, thus resulting in a reasonable target for PC depletion in desensitization therapy (123) or deletion of plasma cells during active AMR (124). Daratumumab is a human IgGκ monoclonal antibody that targets CD38 and induces apoptosis of PC (122, 125) *via* Fcγ receptor-mediated cross-linking (126) and macrophage-mediated phagocytosis (127). In addition to depleting CD38^+^ cells, daratumumab also promotes expansion of memory and naïve T-cells (122), and is approved as monotherapy in patients with multiple myeloma (MM) (122, 125, 128, 129). Isatuximab, is an anti-CD38 mAb also used in the treatment of MM. It induces apoptosis of CD38^+^ cells through Fc-dependent and Fc-independent mechanisms (130), depletes B-lymphocyte precursors (131), and depletes NK cells through direct activation and crosslinking of CD38 and CD16 on NK cells (130). Elotuzumab is an IgG_1_ mAb that targets signaling lymphocytic activation molecule F7 (SLAMF7), also known as CD319, which is highly expressed on MM, NK and other immune cells (132). Elotuzumab was found to activate NK cells and induce apoptosis of SLAMF7^+^ cells *via* both CD16-dependent and CD16-independent mechanisms (132, 133).

There are only anecdotal cases evaluating monoclonal antibodies targeting PC in organ transplantation to prevent or treat antibody-mediated rejection. Daratumumab showed effective desensitization and reversed acute/chronic antibody-mediated rejection (134, 135). In our sensitized NHP model, we reported the effectiveness of daratumumab in combination with an anti-CXCR4 antagonist, plerixafor which mobilizes PC from bone marrow to peripheral blood (135). However, we also reported a possible off-target effect of daratumumab which result in depletion of other CD38 expressing regulatory cells including Treg, Breg, MDSC etc. This feature makes daratumumab attractive for multiple myeloma (122), but could trigger alloimmune responses in transplantation patients. Daratumumab and eculizumab combined therapy reduced *dn*DSAs, improved heart and kidney graft function, and resulted in undetectable circulating PCs. However, class II DSA returned after discontinuing daratumumab therapy (135). The second patient was a highly sensitized recipient who received daratumumab desensitization therapy prior to heart transplantation. After eight weeks, there was found to be reduction in cPRA (98% vs 62%) and class 1 anti-HLA antibodies (35 vs 14) (135). Currently, there is a phase 1 clinical trial to evaluate daratumumab in decreasing circulating antibodies in sensitized recipients awaiting heart transplantation (ClinicalTrials.gov, NCT04088903). There is also a clinical trial to evaluate the safety and efficacy of isatuximab as desensitization therapy in patients awaiting kidney transplantation (ClinicalTrials.gov, NCT04294459). If applied to transplant, these therapies from myeloma field need be carefully evaluated on their off-target effect in a transplantation setting.

### Costimulation Blockade

Rebound of DSA after short-term PI has been reported (136–138). This repletion of PC and DSA would be partially due to an intra-marrow PC repopulation which might be related to PC populations resistant to PI treatment. In the meantime, PC population can expand outside of bone marrow. We observed that the depletion of PC with bortezomib initiated germinal center activation (138). This is probably due to the tightly intertwined network among humoral components. PC may provide a negative feedback loop to Tfh cells (or GC response) since these cell populations compete for similar cytokines/survival factors. Therefore, once one population, in this case PC, disappear then the other cell population (Tfh) is promoted (138). For this reason, targeting T cell help for B cell activation could be a potential strategy for desensitization, especially since the impact of costimulation blockade on humoral responses has been shown in multiple studies (139–141). We and others have reviewed this topic (142–145). It is notable that targeting PC together with costimulation signals successfully prevented the rapid rebound of DSA seen with PI monotherapy (146–149). This suggests that targeting a single humoral component might not be effective in controlling preformed or on-going allo-humoral responses.

### Targeting Mediators/Survival Factor

#### Interleukin-6 Receptor Inhibition

Interleukin-6 (IL-6) is a pleiotropic cytokine produced by many different cell lineages. The membrane-bound IL-6 receptor (IL-6R) is expressed only on hepatocytes and some immune cells (150), but a soluble IL-6R also exists that can bind IL-6 and together this complex can signal through the transmembrane cytokine receptor gp130 (trans-signaling) expressed on nearly all cell types (151). IL-6 is critical for many inflammatory pathways and has a key role in the induction of follicular helper T cells, which direct naïve B cells in the germinal center to differentiate to memory B cells and high-affinity, IgG-secreting plasma cells (152). Accordingly, dysregulated production of IL-6 has been associated with chronic diseases such as diabetes, systemic lupus erythematosus, rheumatoid arthritis, cancer, end-stage renal disease, crescentic glomerulonephritis, and graft versus host disease (153–158). IL-6 has also been associated with deviation of T cells towards a Th17 phenotype, reduction of the proportion of Treg cells, and potentiation of allograft rejection in kidney transplantation (159).

Tocilizumab (Actemra^®^) is a humanized monoclonal antibody with activity against both the membrane and soluble forms of IL-6R approved to treat moderate to severe rheumatoid arthritis, systemic juvenile idiopathic arthritis, polyarticular juvenile idiopathic arthritis, and Castleman’s disease (151). Pharmacologic inhibition of IL-6 signaling is attractive in the context of desensitization strategies, as animal models have shown that this therapy reduces alloantibody responses by inhibition of bone marrow plasma cells and induction of Treg cells (160). Vo et al. recently examined the efficacy of high dose IVIG + tocilizumab in 10 highly sensitized patients who were poorly responsive to high dose IVIG + rituximab (161). This regimen was associated with reduced donor specific antibody number and strength, decreased wait list time, and increased rate of transplantation. No transplanted patients had evidence of antibody-mediated rejection on protocol biopsies. Larger, randomized control trials will be helpful in determining the ultimate value of this treatment given these promising preliminary results.

#### Anti-BAFF Agents

B cell activating factor (BAFF) is a homotrimer and member of the tumor necrosis factor (TNF) family that is found on the cell surface as a transmembrane protein or released in soluble form after cleavage (162). BAFF is secreted by multiple cell types, binds to three separate receptors, and is critical for the maturation of B cells (163). BAFF also acts as a potent B cell activator and is important in B cell proliferation and differentiation. Therefore, blocking this molecule may be essential when targeting allo-B cell response. A monoclonal antibody against BAFF, belimumab (Benlysta^®^), was the first targeted biologic approved for the treatment of systemic lupus erythematosus (164). Belimumab monotherapy was tested for desensitization in kidney transplantation (NCT01025193), but this trial was closed early due to a reported lack of efficacy. Blisibimod is a second anti-Baff agent developed for SLE. It is a fusion protein consisting of four BAFF binding domains. This anti-BAFF agent completed Phase II testing and currently being tested in a Phase 3 trial, CHABLIS-SC1 [(165), NCT01162681]. While considerable progress has been made in the field of desensitization, many potential and untested therapies remain. Other anti-BAFF agents including tabalumab, atacicept, and blisibimod have not been evaluated for desensitization in human trials.

## A Multi-Modal Approach to Desensitization

**Figure 1:**
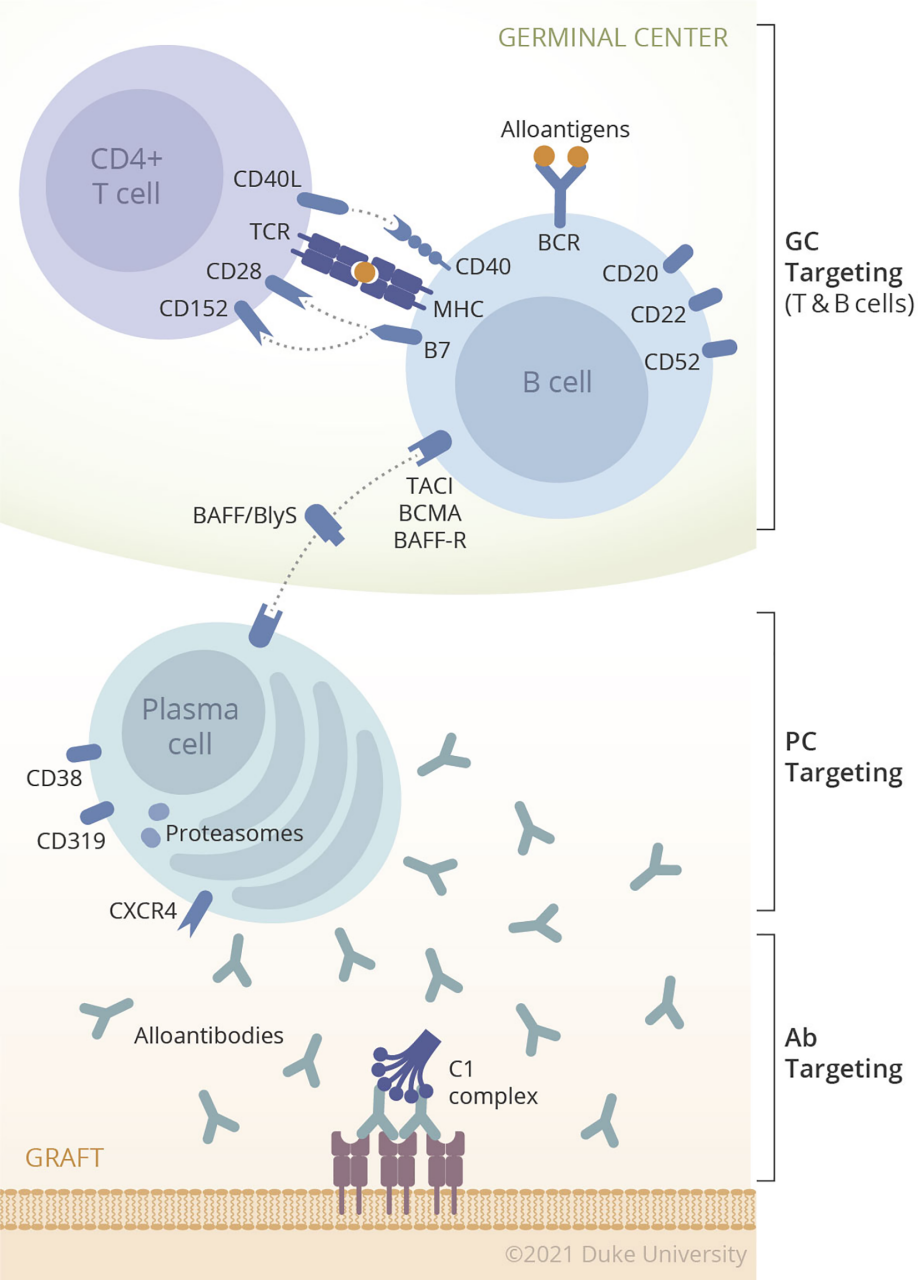
Multiple components of humoral immunity in organ transplantation.

The concept of desensitization has been expanded from only targeting alloantibody (IVIG/IA/plasmapheresis) to instead targeting the upstream sources of antibody such as B cells (Rituximab) and PC (proteasome inhibitor). The conventional desensitization concept, removal of preformed antibody, may prevent hyperacute rejection or acute AMR but without long-lasting impact on humoral alloimmunity. While many desensitization therapies have been tried alone or in combination in animal models and human trials, none yet have solved the barriers to transplantation faced by highly sensitized patients with high titer HLA antibodies. The answer to desensitization may lie in novel therapies not yet tested or those outside the field of transplantation. Therefore, upcoming therapies targeting plasma cells are potentially very attractive. However, considering the previous sensitization events to HLA, allograft could trigger the memory response in sensitized patients. For this reason, targeting each component of humoral response such as alloantibody, B cells, or PC, would tentatively reduce the steady state level of DSA, this would not promote long-term control of humoral response after transplantation. Due to its compensatory mechanism, it would more logical that we develop strategies to desensitizing patients that target multiple steps of DSA production. Fortunately, there are many agents targeting each step of the humoral response as shown in **Figure 1** and **Table 1**. Unfortunately, there will also be too many potential combinations of biologics to permit exhaustive evaluation of each possible combination. Therefore, rational approaches merit testing in a preclinical model before being translated into the clinic.

**Table 1:** Broad Overview of Possible Therapeutics for New Desensitization Regimens.

Drug	Target	Development	Reference
***B Cells***			
Ofatumumab	Anti-CD20	FDA approval for CLL	(166, 167)
Ocrelizumab	Anti-CD20	FDA approval for primary progressive multiple sclerosis	(168)
Ocaratuzumab	Anti-CD20	Clinical trials	(169, 170)
Obinutuzumab	Anti-CD20	FDA approved for CLL	(171)
Blisibimod	Anti-BAFF	Clinical trials	(165)
Tabalumab	Anti-BAFF	Clinical trials	(172–175)
Atacicpet	Anti-APRIL & Anti-BAFF	Clinical trials	(176)
BR3-Fc	Anti-BAFF	Clinical trials	(177, 178)
Belimumab	Anti-BAFF	FDA approval for SLE	(179)
hAPRIL.03A & hAPRIL.01A	Anti-APRIL	Pre-clinical	(180)
Epratuzumab	Anti-CD22	Clinical trials	(181, 182)
Lucatumumab	Anti-CD40	Clinical trials	(172, 183)
Dacetuzumab	Anti-CD40	Clinical trials	(172, 184)
Galiximab	anti-CD80	Clinical trials	(185, 186)
***Plasma Cells***			
Indatuximab ravtansine	anti-CD138	Clinical trials	(172, 187)
Isatuximab	Anti- CD38	Clinical trials	(172, 188, 189)
Moxetumomab	anti-CD22 immunotoxin	Clinical trials	(190)
Siltuximab	IL-6 inhibitors	FDA approval for multicentric Castleman’s disease	(172, 191, 192)
Daratumumab	Anti-CD38	FDA approval for multiple myeloma	(172, 191)
MOR202	Anti-CD38	Clinical trials	(172, 191)
Elotuzumab	Anti-CS1	FDA approval for multiple myeloma	(193)
Milatuzumab	Anti-CD74	Clinical trials	(172)
***T Follicular Cells***			
Pembrolizumab	PD-1 inhibitor	FDA approval for unresectable or metastatic solid tumor	(191, 194)
Nivolumab	PD-1 inhibitor	FDA approval for inoperable or metastatic melanoma	(172, 191, 195–198)
Pidilizumab	PD-1 and DLL1 Inhibitor	Clinical trials	(191)
BGB-A317	PD-1 inhibitor	Clinical trials	(199)
Durvalumab	PD-L1	FDA approval for locally advanced or metastatic urothelial carcinoma	(200–202)
***Ubiquitin-Proteasome Inhibitors***			
IPP-201101	Spliceosomal peptide	Clinical trials	(203, 204)
Marizomib	Proteasome inhibitor	Clinical trials	(203, 205–209)
Delanzomib	Proteasome Inhibitor	Clinical trials	(203, 210)
Oprozomib	Proteasome Inhibitor	Clinical trials	(203)
IPSI-001	Immunoproteasome	Pre-clinical	(115, 203, 211)
ONX-0914	Immunoproteasome	Pre-clinical	(203, 212)
PR-924	Immunoproteasome	Pre-clinical	(203, 213)
RO5045337	Ubiquitin E3 ligase	Clinical trials	(203)
RO5503781	Ubiquitin E3 ligase	Clinical trials	(203)
LCL161	Ubiquitin E3 ligase	Clinical trials	(203, 214)
AEG 35156	Ubiquitin E3 ligase	Clinical trials	(203, 215, 216)
Lenalidomide	Ubiquitin E3 ligase	FDA approval for multiple myeloma and myelodysplastic syndromes	(203)
Pomalidomide	Ubiquitin E3 ligase	FDA approval for relapsed and refractory multiple myeloma	(203)
Ubistatins	19S proteasome	Pre-clinical	(203, 217)
b-AP15	19S *DUBs	Pre-clinical	(203)
P5091	DUBs	Pre-clinical	(203)
P22077	DUBs	Pre-clinical	(203)
WP-1130	DUBs	Pre-clinical	(203)

*DUB - Proteasome-associated deubiquitinases.

## Author Contributions

AC, MM, and DO participated in literature search and wrote the manuscript. BE, JP, and KF participated in writing the manuscript. AJ critically reviewed the manuscript. JK and SK participated in writing and reviewing the manuscript. All authors contributed to the article and approved the submitted version.

## Funding

This work was partially supported by the National Institute of Allergy and Infectious Diseases of the National Institutes of Health as part of the Nonhuman Primate Transplantation Tolerance Cooperative Study Group under U19AI131471 (awarded to SK) and Opportunities Pool Round 13 (awarded to JK). 

## Disclaimer

The content is solely the responsibility of the authors and does not necessarily represent the official views of the National Institutes of Health.

## Conflict of Interest

The authors declare that the research was conducted in the absence of any commercial or financial relationships that could be construed as a potential conflict of interest.
